# Succinate Accumulation Is Associated with a Shift of Mitochondrial Respiratory Control and HIF-1α Upregulation in PTEN Negative Prostate Cancer Cells

**DOI:** 10.3390/ijms19072129

**Published:** 2018-07-21

**Authors:** Anja Weber, Helmut Klocker, Herbert Oberacher, Erich Gnaiger, Hannes Neuwirt, Natalie Sampson, Iris E. Eder

**Affiliations:** 1 Department of Urology, Medical University of Innsbruck, 6020 Innsbruck, Austria; anja.weber@i-med.ac.at (A.W.); Helmut.KLOCKER@tirol-kliniken.at (H.K.); natalie.sampson@i-med.ac.at (N.S.); 2 Institute of Legal Medicine and Core Facility Metabolomics, Medical University of Innsbruck, 6020 Innsbruck, Austria; herbert.oberacher@i-med.ac.at; 3 D. Swarovski Research Lab, Department of Visceral, Transplant Thoracic Surgery, Medical University of Innsbruck, 6020 Innsbruck, Austria; erich.gnaiger@oroboros.at; 4 Oroboros Instruments, 6020 Innsbruck, Austria; 5 Department of Internal Medicine IV-Nephrology and Hypertension, Medical University of Innsbruck, 6020 Innsbruck, Austria; hannes.neuwirt@i-med.ac.at

**Keywords:** prostate cancer, mitochondria, oxidative phosphorylation, acidic tumor microenvironment, Na^+^-dicarboxylate transporter, succinate

## Abstract

The idea of using metabolic aberrations as targets for diagnosis or therapeutic intervention has recently gained increasing interest. In a previous study, our group discovered intriguing differences in the oxidative mitochondrial respiration capacity of benign and prostate cancer (PCa) cells. In particular, we found that PCa cells had a higher total respiratory activity than benign cells. Moreover, PCa cells showed a substantial shift towards succinate-supported mitochondrial respiration compared to benign cells, indicating a re-programming of respiratory control. This study aimed to investigate the role of succinate and its main plasma membrane transporter NaDC3 (sodium-dependent dicarboxylate transporter member 3) in PCa cells and to determine whether targeting succinate metabolism can be potentially used to inhibit PCa cell growth. Using high-resolution respirometry analysis, we observed that ROUTINE respiration in viable cells and succinate-supported respiration in permeabilized cells was higher in cells lacking the tumor suppressor phosphatase and tensin-homolog deleted on chromosome 10 (PTEN), which is frequently lost in PCa. In addition, loss of PTEN was associated with increased intracellular succinate accumulation and higher expression of NaDC3. However, siRNA-mediated knockdown of NaDC3 only moderately influenced succinate metabolism and did not affect PCa cell growth. By contrast, mersalyl acid—a broad acting inhibitor of dicarboxylic acid carriers—strongly interfered with intracellular succinate levels and resulted in reduced numbers of PCa cells. These findings suggest that blocking NaDC3 alone is insufficient to intervene with altered succinate metabolism associated with PCa. In conclusion, our data provide evidence that loss of PTEN is associated with increased succinate accumulation and enhanced succinate-supported respiration, which cannot be overcome by inhibiting the succinate transporter NaDC3 alone.

## 1. Introduction

Tumor cells are known to adapt their metabolism in order to sustain a high proliferative capacity despite conditions of reduced availability of oxygen and nutrients in the tumor microenvironment [1]. Healthy cells usually fulfill their energy demands via oxidative phosphorylation (OXPHOS) preceded by cytosolic catabolism of glucose to pyruvate, which is further oxidized through the electron transfer system including the tricarboxylic acid cycle (TCA) in the mitochondria, yielding ~34 molecules of adenosine triphosphate (ATP). Under hypoxic conditions, cells produce lactate through glycolysis in order to balance the reduced capacity of OXPHOS [2]. It appears that glycolysis is more suitable for rapid cell growth, as energy is (1) provided faster and (2) intermediates from glycolysis can be used to synthesize macromolecules, which are needed for cancer growth (reviewed by [3]). The concept of using metabolic aberrations as targets for diagnostic or therapeutic intervention has recently gained increasing interest. Especially for more advanced stages of prostate cancer (PCa) where therapy resistance remains a major challenge, the development of new treatment options is in high demand.

**Figure 1:**
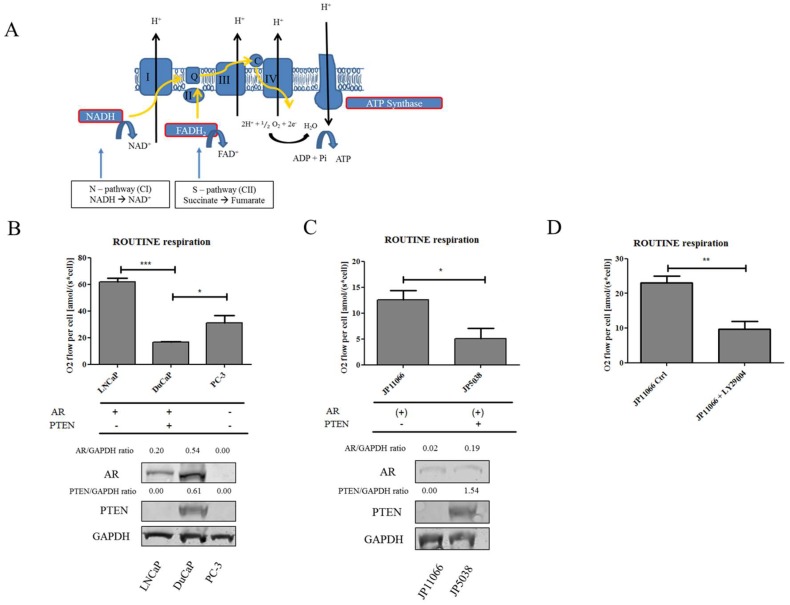
Loss of PTEN results in increased ROUTINE respiration in human and mouse cell lines (**A**) Schematic overview of the electron transfer through the mitochondrial complexes (CI–CII–CIV and CII–CIII–CIV) as part of the electron transfer system. NADH, the substrate for CI, is oxidized to NAD^+^ (N-pathway). Succinate, the substrate for CII, is oxidized to fumarate leading to the reduction of FAD and formation of FADH_2_ (S-pathway). (**B**,**C**) ROUTINE respiration expressed as O_2_ flow per cell was determined by high-resolution respirometry in intact human and mouse cell lines. Androgen receptor (AR) and PTEN expression was validated by western blotting. Glycerinaldehyd-3-phosphate dehydrogenase (GAPDH) was used as internal loading control. Representative blots from three independent experiments are shown. (**D**) ROUTINE respiration expressed as O_2_ flow per cell was determined in intact JP11066 *Pten* KO cells after 24 h treatment with 25 µM PI3K inhibitor LY29004 compared to mock control (DMSO). Data were expressed as mean and SEM of at least three independent experiments. Statistical differences were calculated with *t*-test and indicated with asterisks (*, *p* < 0.05; **, *p* < 0.01; ***, *p* < 0.001).

In a previous study, our group reported major differences in the mitochondrial respiratory capacity of benign prostate and PCa cells. In particular, we showed that PCa cells had a higher respiratory activity than benign cells. Moreover, by scrutinizing the respiratory capacity of pathways through the different segments (complexes CI–CIII–CIV or complexes CII–CIII–CIV) of the electron transfer system (ETS) (Figure 1A), we observed that PCa cells showed a substantial shift towards succinate-supported oxidation via mitochondrial Complex II (CII) compared to benign cells [4]. This shift from NADH- to the succinate-linked pathway (CII) was similarly observed in primary PCa tissue in vivo [5], indicating a functional re-programming of ETS. Notably, succinate, the substrate for CII has been termed an “oncometabolite” [6] and shown to stabilize hypoxia-inducible factor 1-alpha (HIF-1α), leading to a pseudo hypoxic response with activation of HIF-1α target genes such as hexokinase 2. Succinate uptake through plasma and mitochondrial membranes is driven by dicarboxylic acid transporters. The main plasma membrane transporter for succinate is NaDC3 (sodium-dependent dicarboxylate transporter member 3), which has been proposed to be a potential target for cancer therapy [7]. In the present study, we investigated the role of succinate and its transporter, NaDC3, in PCa cells and evaluated the suitability of NaDC3 as a possible target for PCa therapy.

## 2. Results

### 2.1. Loss of PTEN Is Associated with a Shift towards Succinate-Supported Mitochondrial Respiration and an Increase in Intracellular Succinate Levels

There is strong evidence that PCa cells undergo a shift towards the succinate-supported pathway. As a first step, we therefore analyzed oxygen consumption of three human PCa cells using high-resolution respirometry. As shown in Figure 1B, ROUTINE respiration (without uncouplers or inhibitors) measured in intact cells was highest in LNCaP cells, followed by PC-3 and DuCaP cells, which exhibited the lowest rate of ROUTINE respiration. Notably, the oncosuppressor PTEN—which is frequently lost in PCa—is expressed in DuCaP cells but not in LNCaP or PC-3 cells (Figure 1B). To determine whether loss of PTEN has an impact on the cellular respiratory capacity, we next analyzed a murine prostate cell line that was created from a *Pten* knockout (KO) mouse (JP11066) and compared its respiratory activity to that of prostate cells established from a *Pten* wildtype (WT) mouse (JP5038). Indeed, ROUTINE respiration was significantly higher in JP11066 *Pten* KO compared to JP5038 *Pten* WT cells (Figure 1C). PTEN acts as a negative regulator of the phosphatidylinositol-3 kinase (PI3K) pathway. A loss of PTEN expression results in hyperphosphorylation of Akt via PI3K, thereby stimulating cell proliferation and survival [8]. To further evaluate the role of PTEN in the cells’ respiratory activity, we treated *Pten* KO JP11066 cells with the PI3K inhibitor LY294002. As shown in Figure 1D, blocking PI3K activity with LY294002 significantly decreased ROUTINE respiration in *Pten* KO JP11066 cells (Figure 1D).

**Figure 2:**
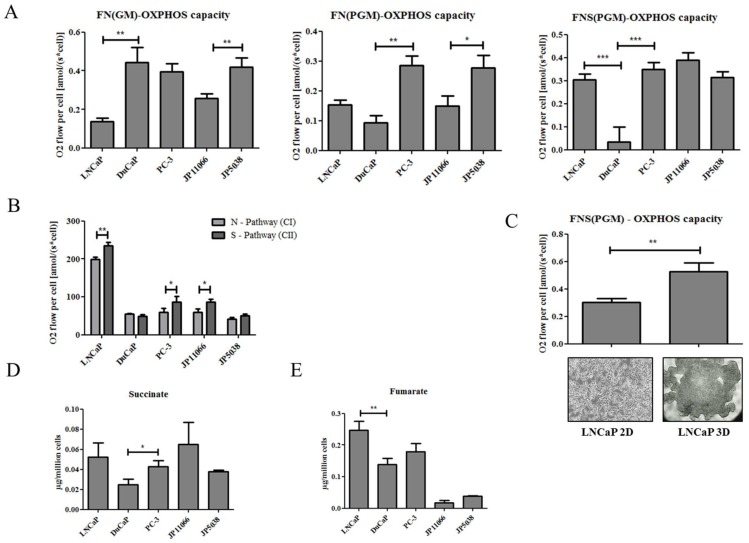
Loss of phosphatase and tensin-homolog (PTEN) is associated with increased capacity for mitochondrial complex II respiration and elevated intracellular succinate levels. Capacities of mitochondrial pathways assessed in permeabilized cells: (**A**) FN(GM) OXPHOS capacity: activation of fatty acid oxidation (F) and NADH linked pathway (N) after addition of glutamate (G) and malate (M), FN(PGM): respiratory capacity after subsequent addition of pyruvate (P), FNS(PGM) OXPHOS capacity after addition of succinate (S). (**B**) N-pathway (complex I, CI) and S-pathway (complex II, CII) respiration. Succinate was added before maximum ETS capacity was reached by addition of uncoupler. Rotenone was added to inhibit CI and assessment of CII-mediated respiration. (**C**) FNS(PGM) OXPHOS capacity determined in LNCaP 3D spheroids and compared to that of LNCaP cells grown in standard 2D culture. Representative images are shown below the graph (magnification 100× (left), 40× (right)) (**D**) Intracellular levels of succinate and fumarate (**E**) were assessed by GC-MS and values expressed as µg per million cells. Data were expressed as mean and SEM. Statistical differences are indicated (*, *p* < 0.05; **, *p* < 0.01; ***, *p* < 0.001).

Next, we permeabilized the cellular plasma membrane to enable a sequential addition of substrates and inhibitors, with each combination stimulating specific mitochondrial pathways separately or in combination (Figure 1A). As depicted in Figure 2A, succinate-mediated respiration (FNS(PGM)-OXPHOS capacity) was significantly lower in DuCaP compared to LNCaP and PC-3 cells. In contrast, FN(PGM)-OXPHOS-capacity (including pyruvate, P, but without succinate) was higher in LNCaP and significantly higher in PC-3 cells compared to DuCaP cells. FN(GM)-OXPHOS-capacity (with glutamate, G, but without pyruvate), on the other hand, was significantly higher in DuCaP compared to LNCaP, and in JP5038 compared to JP11066 (Figure 2A). These data suggest that respiration of PTEN^+^ cells was more activated by the substrates for the N-pathway (CI), while respiration of PTEN^−^ cells was higher for the S-pathway (CII).

Calculating the capacities of the N- and S-pathways separately showed a significant difference of N- versus S-pathway in PTEN^−^ LNCaP, PC-3 and *Pten* KO JP11066 but not in PTEN^+^ DuCaP and *Pten* WT JP5038 cells (Figure 2B). Notably, succinate-supported respiration was further increased when LNCaP cells were cultured as 3D spheroids (Figure 2C). This model has recently been described to more accurately mimic human PCa tissue than 2D cultured cell lines [9]. Since succinate is the substrate fueling the S-pathway, we next determined intracellular succinate levels in the different cell lines by GC-MS. Corresponding with elevated S-pathway capacity, human PTEN negative LNCaP and PC-3 cells indeed exhibited higher levels of intracellular succinate compared to PTEN positive DuCaP cells (Figure 2D), with a significant difference in PC-3 compared to DuCaP cells. Similarly, we found higher levels of intracellular succinate in mouse *Pten* KO JP11066 cells compared to *Pten* WT JP5038 cells (Figure 2D). Additionally, intracellular fumarate, which is formed from succinate in the reaction catalyzed by succinate dehydrogenase, was also significantly higher in LNCaP and higher in PC-3 compared to DuCaP cells (Figure 2E). In the murine cell lines, fumarate levels were generally low with no difference between the two cell lines. Overall, we show here that loss of PTEN is associated with a shift towards succinate-supported S-pathway and an increase in intracellular succinate levels.

### 2.2. Elevated Succinate Levels Are Associated with Increased Lactate Production and Higher Expression of HIF-1α

**Figure 3:**
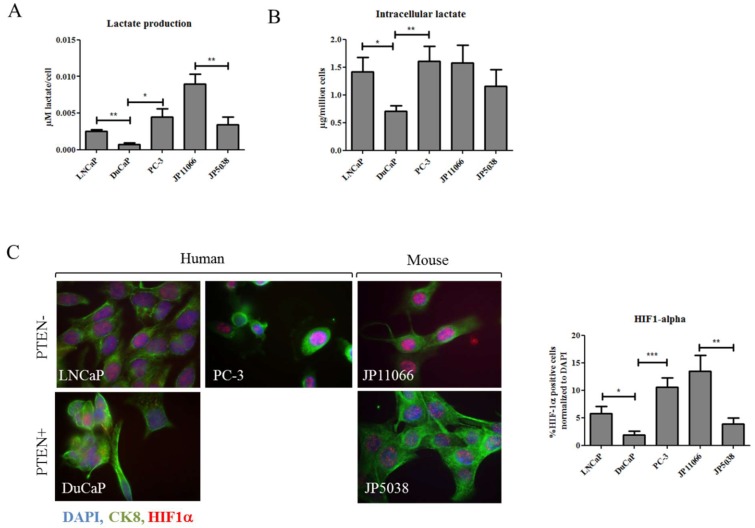
Loss of PTEN is associated with increased lactate production and HIF-1α expression. (**A**) Lactate was measured in the cell culture supernatant using a colorimetric assay and normalized to cell number. (**B**) Intracellular lactate levels were assessed by GC-MS and expressed as µg lactate per 1 million cells. (**C**) Representative images showing immunofluorescent staining for HIF-1α (red) in PTEN negative and positive human and mouse prostate cell lines. To visualize cytoplasm, cells were stained for cytokeratin 8 (CK8, green), and nuclei were stained with DAPI (blue) (magnification 630×). HIF-1α expression was quantified with TissueQuest software and normalized to total cell number as determined by DAPI. Data were expressed as mean and SEM. Statistical differences were indicated in the graphs (*, *p* < 0.05; **, *p* < 0.01; ***, *p* < 0.001).

Loss of PTEN has previously been linked to elevated lactate production and increased glucose consumption [10]. Consistently, we observed that LNCaP and PC-3 cells secreted significantly more lactate than DuCaP cells and lactate secretion was also significantly raised in *Pten* KO JP11066 compared to *Pten* WT JP5038 cells (Figure 3A). In addition, intracellular lactate as determined by GC-MS was significantly higher in LNCaP and PC-3 compared to DuCaP cells (Figure 3B). We also found higher amounts of intracellular lactate in *Pten* KO JP11066 compared to *Pten* WT JP5038 cells although the difference lacked statistical significance. Thus, suggesting that loss of PTEN is not only associated with increased secretion, but also with elevated intracellular accumulation of lactate. Since high lactate levels can induce intracellular acidification, which subsequently increases succinate uptake and leads to a stabilization of HIF-1α [7,11], we examined HIF-1α levels by immunofluorescence. Indeed, HIF-1α expression was significantly elevated in LNCaP and PC-3 cells compared to DuCaP cells (Figure 3C). Furthermore, HIF-1α expression was significantly higher in *Pten* KO JP11066 compared to *Pten* WT JP5038 cells (Figure 3C), suggesting that increased succinate and lactate levels in PTEN negative cells elicit a pseudo-hypoxic regulation by stabilization of HIF-1α.

### 2.3. Knockdown of NaDC3 Expression Is Not Sufficient to Prohibit Succinate Accumulation in Prostate Cancer Cells

**Figure 4:**
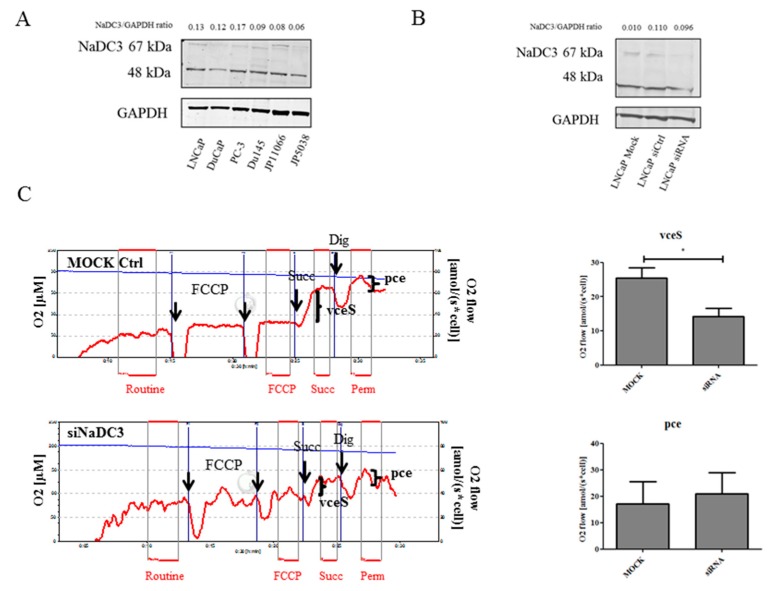
Effects of siRNA-mediated downregulation of NaDC3 on succinate-stimulated respiration. (**A**) Basal NaDC3 protein expression was evaluated in various PCa cell lines via western blotting. GAPDH was used as loading control. (**B**) LNCaP cells were transfected with 50 nM siNaDC3 and lipofectamine 2000. NaDC3 protein levels were determined and compared to a siCtrl and lipofectamine only (Mock) via western blotting. Numbers indicate relative ratios of densitometrical analysis of NaDC3 divided by the reference protein GAPDH. (**C**) Following treatment of LNCaP cells with siNaDC3 or the mock control, succinate-stimulated respiration was measured by high-resolution respirometry. To measure the effect on cell respiration by transporter-mediated uptake, succinate (S) was added to intact noncoupled (FCCP, carbonyl cyanide m-chloro phenyl hydrazine) cells and enhancement of respiration was calculated as S-pathway capacity in viable cells (vceS). Thereafter, digitonin was added to permeabilize the cells and determine stimulation of respiration by extracellular succinate bypassing cellular transporter systems (permeabilized cells, pce). Data were expressed as mean and SEM. Statistical differences were indicated in the graph (*, *p* < 0.05).

Intracellular succinate can originate from the intermediary metabolism, particularly in the TCA cycle, or it can be taken up from the extracellular environment [12]. Uptake of succinate is mainly managed by the plasma membrane transporter NaDC3. The transporter activity is increased in an acidic microenvironment [7]. To investigate the role of NaDC3 in prostate cancer cells, we first determined NaDC3 protein expression in various PCa cell lines. As shown in Figure 4A, NaDC3 expression was higher in LNCaP compared to DuCaP and in *Pten* KO JP11066 compared to *Pten* WT JP5038 cells, indicating an association of NaDC3 expression levels with PTEN expression status. Similarly, NaDC3 mRNA levels were highest in LNCaP, followed by DuCaP and PC-3 cells (Figure S1A). Notably, western blotting revealed that, besides the band for NaDC3 at the expected size of 67 kDa, there was an additional band at the size of approximately 48 kDa in all cell lines. Moreover, we observed another slightly larger band at about 70 kDa in the two mouse cell lines. These additional bands however were not considered for protein quantification. Overall, expression levels were rather moderate in all cell lines tested, indicating that NaDC3 is only weakly expressed in prostate cancer cells. NaDC3 expression was also weak in benign prostate and PCa tissue as determined by immunohistochemistry using the same antibody that was used for western blotting. Kidney tissue, by contrast—which was used as positive control—showed a strong staining for NaDC3 (Figure S1B). We next investigated if downregulation of NaDC3 expression with a small interference RNA (siNaDC3) would influence respiration or viability of PTEN negative LNCaP cells. As shown in Figure 4B, NaDC3 protein levels were reduced by the siRNA compared to mock control and si-control but did not affect mRNA expression (Figure S1C). However, treatment of LNCaP cells with the siNaDC3 resulted in a significant decrease in S-pathway capacity in viable cells (vceS) compared to mock control (Figure 4C). When we looked at the S-pathway capacity in permeabilized cells (pce), on the other hand, we did not see any difference between mock and siRNA treatment (Figure 4C). If blockage of NaDC3 through the siRNA would completely prevent uptake of succinate or if mitochondrial succinate levels maintained by TCA cycle activity were low and limited respiration, we would expect that cell permeabilization should cause a flush of extracellular succinate in siRNA treated cells and a subsequent increase in respiration (pce).

**Figure 5:**
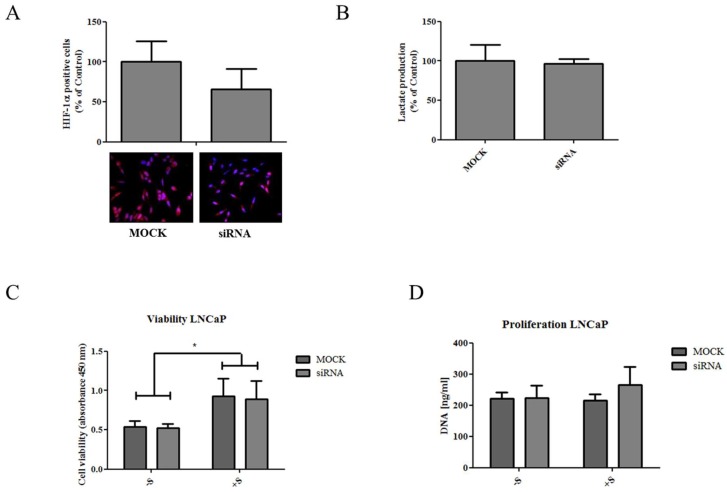
Downregulation of NaDC3 only moderately reduces HIF-1α protein levels and lactate production and does not affect cell viability or proliferation. LNCaP cells were treated with 50 nM siNaDC3 or mock-treated. (**A**) HIF-1α expression was determined by immunofluorescent staining in LNCaP cells after treatment with siNaDC3 (50 nM) or in mock controls. The percentage of HIF-1α positive cells was analyzed using TissueQuest software and normalized for cell number as determined by DAPI staining. Representative images are shown below the graph (magnification 100×). (**B**) Lactate was measured in the cell culture supernatant via a colorimetric assay and expressed as % of Ctrl. (**C**) LNCaP cells were transfected with the siNaDC3. Six hours later, 10 mM succinate (+S) was added. Effects were compared with siNaDC3 treated cells and the mock control cultured in the absence (−S) or presence (+S) of succinate using ANOVA. Cell viability was assessed by WST-1 assay 24 h later and indicated as absorbance at 450 nm. (**D**) Cell proliferation was determined through DNA quantification after staining with SybrGreen. Fluorescence was measured on a Tecan plate reader. Mean DNA amounts and SEM are specified as ng/mL. Statistical differences are indicated (*, *p* < 0.05).

Downregulation of NaDC3 only moderately affected HIF-1α protein levels (Figure 5A) and lactate production (Figure 5B). Since succinate is not only taken up by the cells through the cell membrane but mainly produced intracellularly in the TCA cycle, we next investigated if exogenously added succinate influences cell viability and proliferation. When the cells were treated with 10 mM succinate for 24 h, cell viability was significantly increased in mock control cells (Figure 5C). However, this succinate-induced increase in cell viability could not be reversed by NaDC3 knockdown (Figure 5C). Notably, succinate did not affect cell proliferation and treatment of cells with the siNaDC3 did not affect cell proliferation in the absence or presence of succinate, respectively (Figure 5D). These data suggest that NaDC3 downregulation unlikely affects cell viability and proliferation. The succinate-induced increase in cell viability, on the other hand, is most probably a phenomenon of the WST-1 assay where succinate dehydrogenase activity is measured to estimate cell viability.

### 2.4. Pharmacological Inhibition of Dicarboxylic Acid Transporters Attenuates Succinate Effects and Inhibits Cell Proliferation

**Figure 6:**
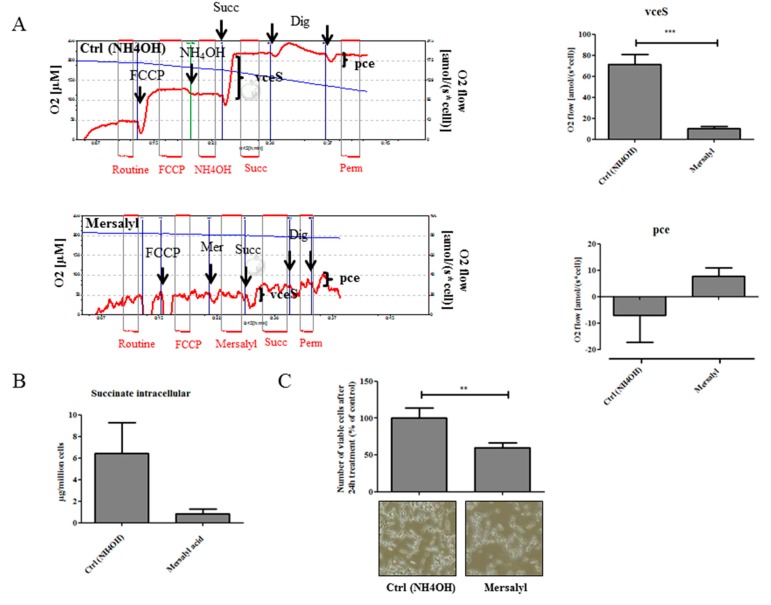
Inhibition of dicarboxylic acid transporters through mersalyl acid decreases succinate-stimulated respiration and cell number. (**A**) LNCaP cells were harvested for measurement of S-pathway capacity in viable cells (vceS). After addition of digitonin to permeabilize the cells, stimulation of respiration by succinate bypassing cellular transporter systems (pce) was measured. To this end, mersalyl acid was added to the O2k chamber as described in material and methods. (**B**) LNCaP cells were treated with mersalyl acid for 72 h (mersalyl acid was added twice after 24 and 48 h) and harvested for the analysis of intracellular succinate with GC-MS and (**C**) the number of viable cells using CASY^®^ cell counter. Representative images of cells treated with mersalyl acid for 24 h and mock control are shown below the graph (magnification 100×). Data are expressed as mean plus SEM. Statistical differences are indicated (**, *p* < 0.01; ***, *p* < 0.001).

Considering these modest effects of NaDC3 downregulation on the metabolic and proliferative activity of LNCaP cells and the rather low NaDC3 expression levels in PCa cells in general, we considered that specifically interfering with NaDC3 alone might not be sufficient to successfully block succinate uptake. We therefore treated LNCaP cells with mersalyl acid, which specifically inhibits dicarboxylic acid transporters by interacting with protein sulfhydryl groups [7]. As shown in Figure 6A, addition of mersalyl acid to the O2k chamber significantly inhibited S-pathway capacity in LNCaP cells (vceS) compared to the mock control (NH_4_OH). After cell permeabilization with digitonin (Dig), pce was markedly decreased in the mock control compared to mersalyl treated cells (Figure 6A). These data suggest that in mock treated cells the whole amount of succinate was able to enter the non-permeabilized cells so that digitonin treatment does not result in any further succinate influx that would yield an increase in respiration. In mersalyl-treated cells, on the contrary, there is still some succinate left in non-permeabilized cells, which then enters the cells after addition of digitonin, resulting in a moderate increase of respiration. In addition, treatment with mersalyl acid over 72 h markedly reduced intracellular succinate levels (Figure 6B) and significantly reduced cell number (Figure 6C). These data suggest that inhibition of all dicarboxylate transporters in general through mersalyl acid, but unlikely the specific blockage of NaDC3 alone, interferes with succinate uptake and S-pathway capacity in PTEN^−^ prostate cancer cells.

## 3. Discussion

Earlier findings by us and others provided considerable evidence that succinate metabolism plays an important role in PCa [4,5,7,13]. Notably, we have previously shown that PCa cells undergo a switch towards succinate-stimulated respiration [4,5]. In particular, we were able to demonstrate significantly increased ROUTINE respiration as well as succinate-induced respiration in LNCaP PCa cells compared to benign prostate RWPE-1 cells [4]. In line with these data, succinate-induced respiration was significantly higher in PCa compared to benign tissue [14]. In the present study, we investigated the mitochondrial respiration capacity of various PCa cell lines and found that loss of PTEN, which is one of the most frequent genetic aberrations in PCa [13], is associated with increased ROUTINE respiration measured in intact cells and higher succinate-stimulated respiration in permeabilized cells. In line with these results, we detected higher intracellular succinate levels in PTEN negative compared to PTEN positive cells, indicating that loss of PTEN may be linked to succinate accumulation. Notably, loss of PTEN and higher succinate levels were also associated with increased HIF-1α expression and elevated lactate production. It was previously shown that succinate uptake is facilitated under acidic conditions, which are provided by high lactate levels. Concurrently, HIF-1α is stabilized and triggers a hypoxia response [7]. Interestingly, a key regulator of this phenomenon is lactate transporter monocarboxylate transporter 1 (MCT1). Inhibiting MCT1 was recently shown to block lactate-induced HIF activation [15]. Our findings show increased HIF-1α expression and elevated lactate levels in PTEN negative compared to PTEN positive PCa cells, suggesting a metabolic switch from the mitochondrial NADH-linked to the succinate-linked pathway. In addition, we have shown that treatment of JP11066 *Pten* KO cells with the PI3K inhibitor LY294002 significantly reduced respiratory activity compared to the mock control. A previous study by Goo et al. has shown that loss of PTEN is associated with increased mitochondrial respiratory capacity and that this was regulated through the Akt/mTOR (mammalian target of rapamycin)/4E-BP1(initiation factor 4E (eIF4E)-binding protein) cascade [8]. In brief, these authors showed that loss of PTEN results in activated Akt, which in turn activates mTOR. Consequently, the downstream target of mTOR, 4E-BP1, is inactivated upon phosphorylation leading to a translocation of respiratory complex components, thereby contributing to increased OXPHOS [8]. This may provide progressive PCa cells lacking PTEN a further growth and survival benefit. Importantly, a further increase in succinate-stimulated respiration and elevated lactate production (data not shown) was measured in 3D LNCaP spheroids compared to conventional 2D culture, further emphasizing the relevance of succinate-linked respiration in PCa cells. One important aspect that has not been addressed in our study is the influence of steroid hormones and the expression of AR on the activation of enzymes for OXPHOS. A recent study by Audet-Walsh et al. showed that AR and mTOR signaling are strongly linked to each other, thereby modulating cellular metabolism [16]. Hence, an influence of AR signaling in the respiratory capacity of human PTEN negative cell lines cannot be excluded. In addition, we want to point out that by using JP11066 and JP5038 mouse cells we intended to particularly outline the impact of PTEN signaling. Regarding *Pten* KO JP11066 cells, it has to be considered that the cells were isolated from mouse prostate tumors but can still represent a heterogeneous cell mixture, possibly also containing non-tumorigenic cells.

The mitochondrial succinate pathway and CII elicit increasing interest as cancer therapeutic targets. Several inhibitors have been developed and tested for their ability to inhibit cancer growth [17,18,19]. Zhunussova and colleagues previously reported the anti-cancer potential of the Na^+^-dependent membrane transporter NaDC3 which is the main carrier for succinate uptake into the cell [7]. NaDC3 is strongly expressed in tissues with a highly active metabolism such as the kidney, liver, brain, and placenta [20,21,22]. Our study revealed a rather weak protein expression of NaDC3 in all PCa cell lines tested, with a trend to higher protein expression in PTEN^−^ human and *Pten* KO mouse compared to PTEN positive cell lines. The human protein atlas further confirms a low expression of NaDC3 in prostate tissue and a high expression in kidney tissue [23]. In this study, immunohistochemical analysis of NaDC3 also revealed a rather low expression in prostate but a very strong expression in kidney tissue. Consequently, transient downregulation of NaDC3 with a siRNA only moderately reduced succinate-stimulated respiration and intracellular succinate levels. Similarly, siRNA-mediated inhibition of the succinate membrane transporter NaDC3 did not affect cell viability or cell proliferation. Exogenously added succinate also failed to affect cell proliferation. Moreover, the succinate-induced increase in cell viability was most probably a phenomenon of the WST-1 assay measurement, which is based on determining succinate dehydrogenase to estimate cell viability. Hence, overall, our data revealed that NaDC3 is only weakly expressed in PCa cells and therefore a downregulation with siNaDC3 did not significantly affect cell proliferation.

On the other hand, treatment of LNCaP cells with mersalyl acid—a more general inhibitor of dicarboxylic acid transporters (in plasma and mitochondrial membranes)—significantly modulated succinate metabolism by reducing intracellular succinate levels as well as succinate-stimulated respiration. Mersalyl acid was formerly used as a diuretic to treat elevated blood pressure and edema in the clinic but has been replaced by less toxic diuretics [24]. While inhibition of NaDC3 in the plasma membrane blocks the extracellular uptake of succinate, mersalyl acid additionally interferes with various mitochondrial metabolite carriers [25,26]. This enables the transport of intracellular metabolites through the mitochondrial membrane to compensate for restricted extracellular succinate uptake. Our finding that extracellular addition of succinate had the same effect on mock and siNaDC3 treated LNCaP cells further revealed independence on the extracellular uptake of succinate to maintain cell viability. Thus, blockage of the plasma membrane transporter NaDC3 alone is most likely not sufficient to reduce cancer cell viability.

Our data provide evidence that loss of PTEN is associated with increased succinate accumulation and enhanced succinate stimulated respiration, which cannot be overcome through inhibiting the succinate transporter NaDC3 alone. Loss of PTEN in PCa is strongly associated with poor prognosis [13]. Thus it is critical to better understand the metabolic consequences of PTEN loss in PCa. Overall, we were able to show that PTEN-deficient cells outline a shift towards complex II. Regarding this phenomenon, it has to be considered that there also might be other oncogenic signals contributing to this metabolic alteration. Nevertheless, a link between PTEN and altered succinate metabolism could be an intriguing aspect in prostate cancer diagnosis, particularly in view of recent reports on the use of hyperpolarized succinate for imaging applications [27].

## 4. Materials and Methods

### 4.1. Cell Lines and Growth Conditions

Human cells: LNCaP and PC-3 prostate cancer cell lines were purchased from the American Type Culture Collection (ATCC, Rockville, MD, USA) and used up to passage 70. LNCaP cells were maintained in DMEM (Lonza, Basel, Switzerland) with 10% fetal calf serum (FCS, Gibco BRL, Life Technologies, Carlsbad, CA, USA), 1% GlutaMAX^TM^ (Gibco BRL, Life Technologies), 1% penicillin/streptomycin (Lonza), 1% sodium-pyruvate (Lonza), and 1% HEPES (Sigma-Aldrich, St. Louis, MO, USA). PC-3 cells were maintained in RPMI 1640 (Lonza) with 10% FCS, 1% penicillin and streptomycin and 1% GlutaMAX^TM^ (Gibco BRL, Life Technologies). DuCaP cells were obtained from Professor J. Schalken (Center for Molecular Life Science, Nijmegen, The Netherlands) and maintained in RPMI 1640 with 10% FCS, 1% penicillin and streptomycin and 1% GlutaMAX^TM^. (Gibco BRL, Life Technologies). The two murine cell lines *Pten* KO JP11066 (*Pten* knockout) and *Pten* WT JP5038 (*Pten* wildtype) were kindly provided by Dr. Jan Pencik (Department of Pathology, Medical University of Vienna, Austria) and cultivated in DMEM F12 (Lonza) supplemented with 10% FCS, 1% penicillin/streptomycin, 10 ng/mL cholera toxin (Sigma-Aldrich), 10 ng/mL human epidermal growth factor (Preprotech, Rocky Hill, NJ, USA), 10 µg/mL bovine pituitary extract (Invitrogen, Carlsbad, CA, USA), 1:100 insulin/transferrin/selenite (Invitrogen), 20 µg/mL ciprofloxacin (Sigma-Aldrich). They were isolated from murine prostates harboring an inducible, prostate-specific deletion of the tumor suppressor gene *Pten* (phosphatase and tensin-homolog deleted on chromosome 10). Transgenic mice were generated as previously described [28,29]. In brief, homozygote deletion of *Pten* was achieved by crossing *Pten*^loxp/loxp^ mice with a ARR2Probasin-Cre transgenic line, in which the Cre recombinase is under the control of a prostate-specific probasin promoter [29]. *Pten* KO JP11066 cells were isolated from a mouse prostate 19 weeks after Cre-mediated gene deletion, while *Pten* WT JP5038 derive from a prostate of a wildtype littermate. All cells were cultivated at 37 °C in a humidified atmosphere with 5% CO_2_.

### 4.2. High-Resolution Respirometry (HRR)

Mitochondrial respiration was measured at 37 °C in a two chamber respirometer Oroboros Oxygraph-2k (O2k; Oroboros Instruments, Innsbruck, Austria). Data acquisition and analysis was performed using DataLab software (Oroboros Instruments, Innsbruck, Austria). In brief, cells were trypsinized (Trypsin/EDTA, Lonza), washed with PBS (Lonza) and re-suspended in 5 mL mitochondrial respiration medium (MiR05: 0.5 mM EGTA, 3 mM MgCl_2−_6H_2_O, 60 mM lactobionic acid, 20 mM taurine, 10 mM KH_2_PO_4_, 20 mM HEPES, 110 mM D-sucrose, 1 g/L fatty acid-free bovine serum albumin) to obtain a concentration of 2.5 × 10^5^ cells per mL. The titration protocol was used with some minor modifications as previously described [30]. Plasma membrane permeabilization was performed by titration of 1 µL digitonin (8.1 mM) until ROUTINE respiration was decreased by half. Then, octanoyl-carnitine (0.2 mM) and malate (2 mM) were added and fatty acid ß-oxidation (FAO) linked oxidative phosphorylation measured after the titration of ADP (1 mM; F-OXPHOS). Subsequently, glutamate (G; 10 mM) and pyruvate (P; 5 mM) were added as a source of NADH which is linked to respiratory CI for electron entry into the Q-junction, for measurement of FN(GM) and FN(PGM)-OXPHOS-capacity. Cytochrome *c* (10 μM) was applied to control for mitochondrial outer membrane integrity, followed by the addition of succinate (S; 10 mM) to assess FNS-linked respiration. Uncoupling was performed by stepwise titration of carbonyl cyanide m-chloro phenyl hydrazine (FCCP or CCCP, titration in 2 μM steps until maximum FNS–ET (electron transfer)-capacity was reached). S-ET-capacity was measured after subsequent CI inhibition by rotenone (0.5 μM), which inhibits the F-and N-pathways. Finally, CII and CIII were inhibited by addition of malonate (5 mM) and antimycin A (2.5 μM) to measure and correct for residual oxygen consumption. Respiratory flow, *I*_O2_, was expressed as amol O_2_·s^−1^·cell^−1^, equivalent to pmol O_2_·s^−1^·10^−6^ cells [4]. To investigate succinate uptake in the presence of mersalyl acid, LNCaP cells were added to the O2k chamber at a concentration of 2.5 × 10^5^ cells per mL in MiR05 respiration medium. After stabilization of ROUTINE respiration of intact cells, FCCP (titration in 2 μM steps until maximum ET-capacity reached) was added to stimulate respiration. Then, 100 µM mersalyl acid (dissolved in ammonia solution) or 25% ammonia solution (NH_4_OH, control), followed by 10 mM succinate were added. Finally, plasma membrane permeabilization was performed using digitonin as described above. To assess ROUTINE respiration after PI3K inhibition, JP11066 cells were treated with 25 µM LY29004 or control (DMSO) which was added to the medium 24 h before cells were harvested and high resolution respirometry measurement was performed as mentioned above.

### 4.3. Cell Viability Assay

Cell viability was assessed using a WST-1 assay which is based on cleaving the tetrazolium salt WST-1 to formazan through succinate dehydrogenase (Roche, Basel, Switzerland). Briefly, cells were incubated with the WST-1 reagent (1:10) for 1h and then absorbance was measured on a Chameleon 5025 (HVD Life Sciences, Vienna, Austria) at 450 nm.

### 4.4. Lactate Production

To estimate lactate production, lactate was determined in 20 µL cell culture supernatant using a colorimetric assay and normalized to cell number as previously described [31]. This assay is based on the conversion of lactate to pyruvate by lactate dehydrogenase (LDH) thereby reducing NAD^+^ to NADH, resulting in the reduction of the colorimetric dye P-iodonitrotetrazolium violet INT to INTH, which is then measured at 450–520 nm. To analyze lactate production in mock and siNaDC3 treated cells, we seeded equal amounts of LNCaP into a 6-well plate (250,000 cells/well) and measured lactate in 20 µL of the supernatant 24 h afterwards. 

### 4.5. Proliferation Assay

Cells were seeded in 96-well plates (black plate, clear bottom) in phenol red-free media. Subsequently the medium was aspirated and cells lysed by freezing at −80 °C. Before DNA quantification, 200 μL CyQuant lysis reagent (Invitrogen) containing SybrGreen (final dilution 1/1000) was added. Cells were incubated for 30 min at 37 °C and fluorescence measured on a Tecan Genius Pro (Tecan Trading AG, Männedorf, Switzerland). Intensity of cell-free wells was subtracted as background. DNA concentration of test samples was calculated based on a standard curve generated from known concentrations of lambda DNA. Cell numbers were determined on a CASY^®^ cell counter according to the manufacturer’s protocol (Schärfe System GmbH, Reutlingen, Germany). In particular, the number of viable cells were assessed using the following parameters: capillary: 150 µm; peak diameter: 11.55 µM; size scale: 0–50 µm; aggregation correction: auto; normalization cursor: 7.57–50 µm; evaluation cursor: 11.52–50 µm; dilution factor: 101.0; *x*-axis scaling: 50 µm.

### 4.6. Real-Time Quantitative PCR (qPCR)

RNA isolation, cDNA synthesis, and qPCR were performed as described previously [32]. TaqMan gene expression assays for quantification of SLC13A3 (NaDC3) (Hs00955744_m1) was purchased from Life Technologies. Fold change in gene expression was determined using the mathematical model ratio 2^−ΔΔ*C*t^. Values were normalized to the mean of the housekeeping gene HMBS (Hs00609297_m1, hydroxymethyl-bilane synthase).

### 4.7. Immunofluorescent Staining

Cells were seeded onto glass cover slides and allowed to attach for 24 h. Medium was removed and cells fixed with 4% paraformaldehyde for 10 min. After fixation, cells were washed with PBS and permeabilized with 0.2% Triton^®^-X-100 (Serva, Heidelberg, Germany) for 5 min. Subsequently, cells were incubated in StartingBlock^TM^ (PBS) blocking buffer (ThermoFisher Scientific, Waltham, MA, USA) for 1 h at room temperature. The following primary antibodies were added for 1 h at room temperature: rabbit anti HIF-1α (dilution 1:50; EP1215Y, Abcam, Cambridge, UK) and chicken anti cytokeratin 8/18 (dilution 1:100; Sigma-Aldrich). After removal of primary antibodies, cover slides were washed with PBS and secondary antibodies added: goat anti rabbit 555 (1:200, Invitrogen) and donkey anti mouse 488 (1:200, Invitrogen). Subsequently, slides were mounted in Vectashield mounting medium containing DAPI (Vector Laboratories, Burlington, CA, USA). Cells were visualized using fluorescent microscopy on a Zeiss Axio Imager Z2. HIF-1α staining intensity was scored using TissueQuest software (TissueGnostics, Vienna, Austria) and normalized to DAPI positive cells.

### 4.8. Immunohistochemistry

Immunohistochemical staining of NaDC3 was performed on a tissue micro array (TMA) consisting of paraffin-embedded human prostate benign and cancer tissue as well as kidney tissue that was used as a positive control. The use of the archived samples was approved by the ethics committee of the Medical University Innsbruck. Tissue was counterstained with hematoxylin. NaDC3 antibody (Proteintech, Rosemont, IL, USA) was used at a concentration of 1:40 and immunohistochemistry (IHC) was performed on a Discovery-XT staining device (Ventana, Tucson, AZ, USA) using the standard IHC protocol and antigen retrieval at pH 9 (CC1). Representative images were taken with an Olympus BX53 (Olympus, Shinjuku, Japan).

### 4.9. Western Blotting

Cells were lysed with 100 µL NuPage LDS sample buffer (1.5× in PBS, ThermoFisher Scientific) and sonicated. Gel electrophoresis was performed at 150 V for 1.5 h in NuPage MOPS (3-(N-morpholino) propane sulfonic acid) SDS Running buffer (ThermoFisher Scientific). Transfer was performed using 35 V for 1:35 h in NuPage Transfer buffer (ThermoFisher Scientific). Blots were incubated in StartingBlock^TM^ (PBS) blocking buffer (ThermoFisher Scientific) for 1 h at room temperature. The following primary antibodies were used: rabbit anti AR PG-21 (androgen receptor, dilution 1:500, Merck-Millipore, Burlington, CA, USA), rabbit anti PTEN (phosphatase and tensin homologe, 1:1000, Cell Signaling Technology Inc., Danvers, MA, USA), mouse anti GAPDH (glycerinaldehyd-3-phosphat-dehydrogenase, dilution: 1:50,000, Merck-Millipore), and rabbit anti NaDC3 (dilution: 1:250, Proteintech). Membranes were incubated with fluorescently-labelled secondary antibodies (Molecular Probes, Eugene, OR) and subsequently scanned and quantified using the Odyssey infrared imaging system (LiCor Biosciences, Lincoln, NE, USA).

### 4.10. Transient Inhibition of NaDC3 with Small Interference RNA (siRNA)

Adherent cells were transfected with 50 nM siNaDC3 (NaDC3 siRNA human, Szabo Scandic, Vienna, Austria) or 50 nM of ON TARGET plus non targeting pool (siCtrl, ThermoFisher Scientific,) using lipofectamine 2000 transfection reagent (ThermoFisher Scientific) for 6 h. MOCK treated cells were incubated with lipofectamine solution only.

### 4.11. Quantitative Analysis of Intracellular Succinate, Fumarate, and Lactate by Gas Chromatography-Mass Spectrometry (GC-MS)

2 × 10^6^–5 × 10^6^ cells (depending on availability) were washed with ice cold PBS and resuspended in 150 µL 10 mM phosphate buffer and 850 µL ice cold ethanol absolute added. Cells were sonicated and subjected to a rapid freeze–thaw procedure (30 s in liquid nitrogen followed by a quick thaw at 98 °C). Cells were then centrifuged at 13,200 rpm for 10 min. Supernatant was collected for quantitative analysis. Calibration was accomplished with methanolic standard solutions and nonadecanoic acid as internal standard. Succinic acid, fumaric acid, nonadecanoic acid, pyridine, methoxyamine hydrochloride, *N*-methyl-*N*-trimethylsilyl-trifluoroacetamide (MSTFA) were obtained from Sigma-Aldrich. Dried extract or the dried metabolite standard mix was derivatized to its (Meox-) TMS-derivatives through 2 h reaction with 30 µL of 20 mg/mL methoxyamine hydrochloride solution in pyridine, followed by a 1 h reaction with 60 µL of MSTFA, both at 60 °C. The GC-MS system consisted of a HP7890 GC device with a HP5975C inert XL mass-selective detector (Agilent Technologies, Palo Alto, CA, USA). A DB-XLB column (30 m × 0.25 mm i.d. × 0.25 µm film thickness, J&W Scientific, Folsom, CA, USA) was used for chromatographic separation. The carrier gas was helium with a flow rate of 1.0 mL/min. Injection volume was 1 µL (splitless), injection temperature was 250 °C. The temperature program was as follows: 50 °C, hold 2 min; heat to 310 °C with 10 °C/min, hold for 10 min. MS was done in electron impact mode (70 eV) scanning from 50 to 800. Mass spectral data were recorded on a personal computer with the HP MS ChemStation software G1034C version D01.00 (Agilent Technologies).

### 4.12. 3D Hanging Drop Cell Culture

Three-dimensional spheroids were established as described previously [9]. Briefly, cells were seeded into a 96 well Perfecta3D^®^ hanging drop plate (Sigma-Aldrich) at a cell number of 7500 cells per drop (40 µL). Spheroids were pooled and treated with 200 µL Trypsin/EDTA (Lonza) for 10 min. The enzymatic reaction was stopped with 200 µL Trypsin inhibitor (Sigma-Aldrich). After centrifugation, 1.25 × 10^6^ cells were resuspended in 5 mL MiRO5 respiration buffer and respiration was measured under the conditions described above. 

### 4.13. Statistical Analysis

Statistical differences were calculated via *t*-test or in case of non-Gaussian distribution non-parametric tests using GraphPad Prism5 (GraphPad Software, La Jolla, CA, USA). In case of comparison of more than two groups in one single statistical test (e.g., Figure 5C) ANOVA was used. Compared groups are indicated in the figures and/or figure legends. Significant differences are denoted as follows: *, *p* < 0.05; **, *p* < 0.01; ***, *p* < 0.001. Since the study was of exploratory nature no pre-defined procedure for statistical analyses existed. Hence, we tested for each single experiment for statistical significance and did not perform correction for multiple testing; in particular, we did not change the overall predefined level of significance (alpha) of 5%. Data are presented as mean plus standard error of the mean (SEM) from at least three independent experiments unless otherwise stated.

## Supplementary Materials

Supplementary materials can be found at http://www.mdpi.com/1422-0067/19/7/2129/s1.

## Abbreviations

PCa	Prostate cancer
HIF-1α	Hypoxia-inducible factor 1 alpha
PTEN	Phosphatase and Tensin homologe
NaDC3	Sodium-dependent dicarboxylate transporter member 3
siRNA	Small interference ribonucleic acid
siCtrl	Small interference control
OXPHOS	Oxidative phosphorylation
ATP	Adenosine triphosphate
CI:II, III, IV	Complex I, II, III, IV
ATCC	American Type Culture Collection
FCS	Fetal calf serum
DMEM	Dulbecco’s modified Eagle’s medium
RPMI	Roswell Park Memorial Institute
O2k	Oxygraph-2k
FCCP,CCCP	Carbonyl cyanide m-chloro phenyl hydrazine
LDH	Lactate dehydrogenase
NAD	Nicotinamide adenine dinucleotide
NADH	Nicotinamide adenine dinucleotide hydride
INT	P-iodonitrotetrazolium violet
INTH	P-iodonitrotetrazolium violet hydride
WST-1	Water soluble tetrazolium-1
DNA	Deoxyribonucleic acid
MOPS	3-(*N*-morpholino) propane sulfonic acid
PBS	Phosphate buffered saline
AR	Androgen receptor
GAPDH	Glycerinaldehyd-3-phosphat-dehydrogenase
MSTFA	*N*-Methyl-*N*-trimethylsilyl-trifluoroacetamide
GC-MS	Gaschromatography-mass spectrometry
3D	3-dimensional
MiR05	Mitochondrial respiration medium 05
SEM	Standard error of the mean
WT	Wildtype
KO	Knockout
FAD	flavin adenine dinucleotide
FADH	flavin adenine dinucleotide hydride
S-ET	Succinate–electron transfer
FN(PGM)	Fatty acid oxidation, N-pathway (pyruvate, glutamate, malate)
FNS(PGM)	Fatty acid oxidation, N-pathway, S-pathway (pyruvate, glutamate, malate)
VceS	Viable cells Succinate
Pce	Permeabilized cells
Succ	Succinate
Dig	Digitonin
NH_4_OH	Ammonia solution
Na	Sodium
RNA	Ribonucleic acid
IHC	Immunohistochemistry
qPCR	Quantitative polymerase chain reaction
MCT1	Monocarboxylate transporter 1
mTOR	Mammalian target of rapamycin
PI3K	Phosphatidylinositol-4,5-bisphosphate 3-kinase
4E-BP1	Initiation factor 4E (eIF4E)-binding proteins
AMACR	p63/α-methylacyl-CoA racemase
TMA	Tissue microarray
kDA	Kilodalton
DMSO	Dimethyl sulfoxide
