# Supplementary figures and images for: Succinate Accumulation Is Associated with a Shift of Mitochondrial Respiratory Control and HIF-1α Upregulation in PTEN Negative Prostate Cancer Cells
Source: Int J Mol Sci. 2018 Jul 21;19(7):2129. doi: 10.3390/ijms19072129 (PMC6073160; doi:10.3390/ijms19072129)

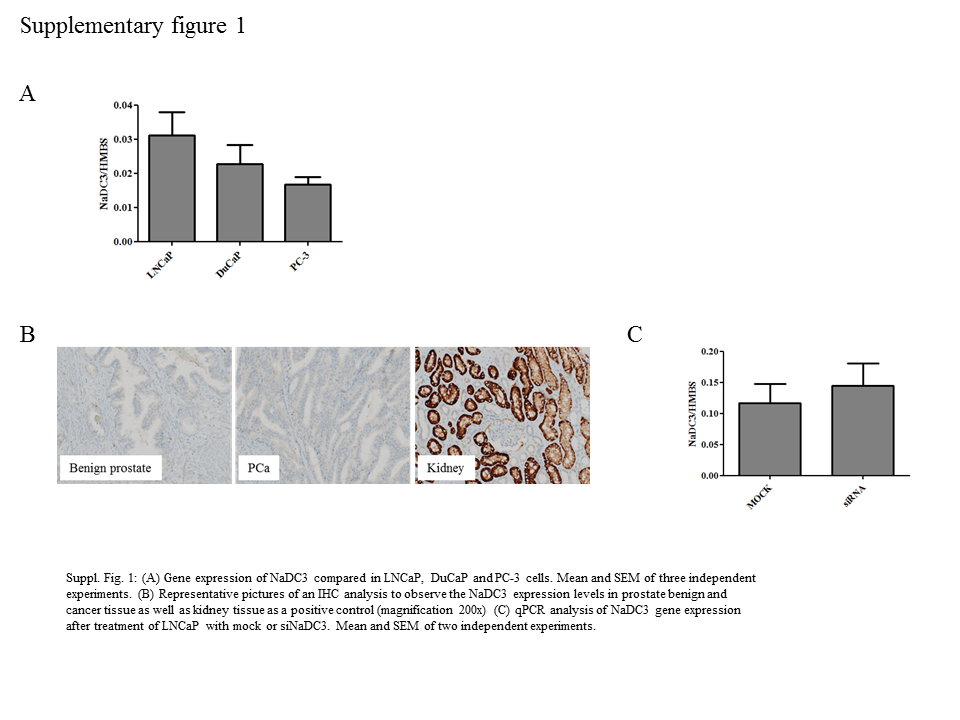

Supplement: Supplementary file 1 [file ijms-19-02129-s001.jpg]
